# Evaluation of clinical trial of atopic dermatitis by a topical cream containing the extracts from photosynthetic bacteria, *Rhodobacter sphaeroides*

**DOI:** 10.1186/s13568-015-0133-4

**Published:** 2015-08-12

**Authors:** Nam Young Kim, Jeong Sub Cho, Hyeon Yong Lee

**Affiliations:** Department of Medical Biomaterials Engineering, Kangwon National University, Kangwon University-ro 1, Chuncheon, Kangwon 200-701 South Korea; DooSan EcoBizNet, Soyanggang-ro 56, Chuncheon, Kangwon 200-161 South Korea; Department of Food Science and Engineering, Seowon University, Musimseo-ro, Cheongju, Chungbuk 361-742 South Korea

**Keywords:** Atopic dermatitis, Clinical research, Photosynthetic bacteria, *Rhodobacter sphaeroides*

## Abstract

The photosynthetic bacteria *Rhodobacter sphaeroides* has been studied as a functional food source; however, in this clinical study, we report for the first time its use as a treatment for atopic dermatitis. Topical cream containing 10% (v/v) extract was demonstrated to have the ability to reduce skin moisture content loss and pruritus by 27.82% in clinical trials for atopic dermatitis compared with controls. In particular, there were statistically significant differences in the pH and temperature changes of the skin, skin firmness and general skin appearance. Changes in the skin pH were measured as 4.83, and there was a 3.37% change in temperature after 4 weeks of treatment. It was also found that there were great differences in wrinkle states according to the grading scale of patients before and after treatment with topical cream. Therefore, these results strongly suggest that extracts from photosynthetic bacteria can be employed to soothe atopic irritation as a new cosmetic bioresource.

## Introduction

Due to atmospheric pollution and changes in residential environments resulting from the rapid industrialization of modern society, the number of patients with atopic dermatitis has been recently increasing. Atopic dermatitis is a skin disease that may occur in any age group that is characterized by severe itch resulting from chronic inflammatory eczema. Genetic factors are strongly related to this disease, and stress, changes in external environments, and infection are major causes of atopic dermatitis aggravation. A major symptom of atopic dermatitis is severe itch, and this disease induces abrasions, erythematous eruption, blisters, and various lesions (Shultz and Hanifin 2002). Because the cause of the symptoms for atopic dermatitis has not been clearly established (Bos et al. 1992), studies of the cause have been unceasingly conducted. Although the management of environmental conditions that may be problematic and drug treatment centering on steroids and antihistamines are used as major treatment methods (Park et al. 2002), the symptoms of this disease are only temporarily relieved, and the aggravation and relief of symptoms are repeated. In addition, adverse affects to some external application products are emerging. Therefore, recent studies have mainly been conducted using products for external application containing oriental medicinal extracts with natural materials that enhance moisturizing effects as therapeutic agents for atopic dermatitis (Hong and Jung 2014). Although clinical effects from extracts from various natural products used as atopic dermatitis treatment materials have been demonstrated, no clear solution has been presented until now. Therefore, among the microorganisms that may be easily produced due to their ability to be cultured in large numbers, we aimed to identify the effects of the photosynthetic bacteria *Rhodobacter sphaeroides* on atopic dermatitis.

There have been reports indicating that the facultative anaerobic photosynthetic bacteria *Rhodobacter sphaeroides* produces SOD (superoxide dismutase) (Kim and Lee 2004). SOD is an anti-oxidant enzyme that has been reported to convert harmful oxygen radicals in the body into H_2_O_2_, and it has oxidation inhibitory functions via its conversion into water and oxygen by catalase to remove harmful active oxygen in vivo, thereby protecting living bodies (Cho and Choi 2013; McCord and Fridovich 1969). Although diverse studies on aging and disease have been conducted using eukaryotic organisms as materials, studies on microorganisms existing in the natural world in large numbers are insufficient. In addition, although photosynthetic bacteria have the abovementioned effects due to their ability to generate hydrogen, they are mainly studied for their function in alternative clean energy (Lee 1986), for use as materials in environmental industries such as livestock manure treatment and waste water purification, and for utilization as foods because they contain large amounts of nutrients (Lee and Lee 2000). In addition to studies on photosynthetic bacteria performed thus far, we utilized the established strengths of photosynthetic bacteria i.e., their production of SOD and catalase and their rich nutrients, to explore new functions of photosynthetic bacteria in relieving skin ailments such as atopic dermatitis by supplying nutrients to the skin and removing harmful active oxygen from the body with the view of preparing a new horizon for photosynthetic bacteria. In addition, we conducted clinical trials for the first time using a formulation for atopic dermatitis to identify the value of photosynthetic bacteria for the treatment of this disease.

## Materials and methods

### Preparation of a topical cream for clinical trials

To first make extracts of photosynthetic bacteria, *Rhodobacter sphaeroides* (ATCC, 21286™, Manassas, VA, USA) was grown with culture medium (ATCC^®^ Medium 550: R 8 A H medium, ATCC, Manassas, VA, USA) and a 20 W fluorescent lamp in a five liter glass flask at pH 6.8 and 30°C by agitating at 100 rpm. After 5 days of cultivation, the cells were collected from the culture broth and broken by a sonicator (AUG-R3-900, Asia Ultrasonic, Bucheon, Korea) for 1 h. Then, the supernatant was filtered by filter paper (5C 110 mm, ADVANTEC, Dublin, CA, USA), and the filtrate was used as a photosynthetic bacteria extract by mixing 10% (v/v) of the extract with a basal formulation of a topical cream as described in Table 1 for a test topical cream.

**Table 1:** The formulation of a topical cream used for clinical trials

Classification	Materials	Content (%)
*Rhodobacter sphaeroide* extracts	Culture medium	10
Water surface region	DI-water	64.5372
	EDTA-2NA	0.018
	Glycerin	3.6
	1.3-BG	1.8
	Keltrol-F	0.045
Thickner I	Carbopol # 940	0.09
Antiseptic	D-M	0.18
	D-P	0.09
Oil I	Stearic acid	0.9
	Kalcol 6870	0.9
	Kcalcol 8688	0.72
	GMS 105	0.9
	Arl 165	0.27
	Mango buffer	0.27
	Myrj 52s	1.08
	PhytoSqualane	1.8
	CIO	2.7
	Jojoba oil	2.7
	LP 70	1.8
	DC 200/100CS	0.45
	Phytoshpingosine	0.135
	Cholestrol	0.45
	PC95	0.045
Oil II	Borage seed oil	0.27
	DC345	1.8
Extract	Germall 115	0.18
	Vitamin E acetate	0.45
	Allantoin	0.09
	DL-panthenol	0.18
	Licollice BG 100	0.18
	SC-glucan	0.9
	Niacinamide	0.0009
	Guava Ext.	0.0009
	KXDUW0028	0.162
pH controller	TEA	0.126
Thickner II	Rheocare ATH	0.18

For more detailed processing for making the topical cream, the water phase was heated to 80–85°C to become transparent. Afterward, a carbopol #940, as a thickner I was added. A preservative, oil part I, and 10% (v/v) of photosynthetic bacteria extract were placed in the oil part in order and heated to 85–90°C. Afterward, after confirming that oil part I was completely melted, oil part II was added at 80°C. The oil part was continuously mixed until the oil phase was completely dissolved with a paddle mixer at 900–1,000 rpm. After confirming that the oil part content was completely dissolved, the oil part was added to the water phase at 75–80°C to conduct the 1st emulsification. Afterward, the solution was homogenized 3 min using a homo mixer at 4,000–4,500 rpm. When the homogenization was completed, the extract and a fragrance was added to the solution at 55°C, and the solution was again homogenized for 5 min using a homo mixer at 4,000–4,500 rpm. A pH conditioning agent was added at 45–50°C, and the solution was again homogenized for 3 min using a homo mixer at 3,500–4,000 rpm. Finally, Rheocare ATH was added to the solution, and the solution was homogenized for 5 min using a homo mixer at 3,500–4,000 rpm to make the formulation.

As a negative control, the cream containing the same basal formulation in Table 1 without only the photosynthetic bacteria extract was used since this formulation is commonly used for the base of most topical creams in Korea (Yu et al. 2004). For a positive control, commercial product (expressed as a control product in this work), a Atopalm^®^ mile cream (ATOPALM, Daejeon, Korea) was used in the experiments, and the composition of major components was as follows: glycerin, propanediol, myristoyl, capric tryglyceride, polyglyceryl-10 distearate, glyceryl stearate, cetearyl alcohol, grape seed oil (3%, v/v), jojoba seed oil, Portulacae Herba extract (5%, v/v), sorbitan stearate, olive oil, hydrogenated vegetable oil, phytosterol, sodium hyaluronate (1%, w/v), tocopheryl acetate, allantoin, stearic acid, carbomer, arginine, dimethicone, 1,2-hexanediol, and caprylyl glycol, tropolone, etc.

### Evaluation of the clinical efficacy for atopic dermatitis

#### Patients

The efficacy of the formulation for atopic dermatitis was examined among males and females between two and 40 years old who had itching symptoms due to xeroderma. The detailed information of the patients in the clinical trials is shown in Table 2. In the clinical trials, the relevant test product was applied to dry areas before the water was dried out after washing the face two times per day for 4 weeks. The water content of the test product, the amount of trans-epidermal water loss, skin temperature, skin pH, VAS, and SCORAD Index (atopic dermatitis symptom index) were evaluated similarly to previous studies (Young and Choi 2005; Yu et al. 2004).

**Table 2:** Groups of the patients participating in the clinical trials

Age	Number of people	Percent (%)
1–9	22	47.8
10–19	9	19.6
20–29	10	21.7
30–39	5	10.9

### Measurement of the skin moisture content and trans-epidermal water loss

Skin moisture content was measured from the skin of test subjects who used the test formulation for 0–4 weeks with a corneometer (GmbH, Cologne, Germany) (Yu et al. 2004) by placing the corneometer probe into contact with the skin, and the average value of five measurements was used. The amount of trans-epidermal water loss was also measured from dry areas of the skin of test subjects who used the photosynthetic bacteria formulation for 0–4 weeks using a Vapometer (Delfin Technologies Ltd, Kuopio, Finland), which measures the amount of trans-epidermal water loss based on the Fick’s law for diffusion, and lower values indicated better skin conditions.

### The changes of pH and temperature of the patient skin

Skin pH was measured with a skin pH meter (PH905, Courage-Khazaka electronic GmbH, Cologne, Germany) because the acid mantles in the skin play the role of antibiotic defensive barriers. Therefore, if the pH of the skin increases, the possibility of skin inflammation due to various bacteria also increase. Skin temperature was also measured with a skin thermometer (ST500, Courage-Khazaka electronic GmbH, Cologne, Germany), which is a device used to measure skin temperature by sensing infrared rays emitted from the atopic dermatitis areas of the skin.

### General high-resolution photograph acquisition

Photographs of atopic dermatitis regions of test subjects were taken before and after treatment (for 4 weeks) with the topical cream by a high-resolution camera (D3300, Nikon, Tokyo, Japan).

### Measurement of Visual Analogue Scale (VAS) for the relief of pruritus

The test subjects were instructed to mark the degree of itch they felt on a 10 cm line by indicating higher degrees with longer lines, and the lengths marked were measured. VAS evaluation for pruritus was conducted by comparing the lengths of the lines indicating the degree of pruritus before and after treatment with the topical cream (Hong et al. 2008).

### Measurement of the Scoring Atopic Dermatitis (SCORAD) index

The SCORAD index (Stalder and TaÏeb 1993) was modified to evaluate the areas of atopic dermatitis symptoms and the degree of the symptoms in the head, trunk, arms, and legs. This experiment was conducted by dermatologists, and subjective symptoms were evaluated by medical examinations through interviews.

### Statistical analysis

All experimental data were performed in triplicate and processed by two-way ANOVA using the software Statistical Analysis System (SAS). The minimum difference in significance level was set to *p* < 0.05.

## Results

### Measurement of the water content of skin

**Fig. 1:**
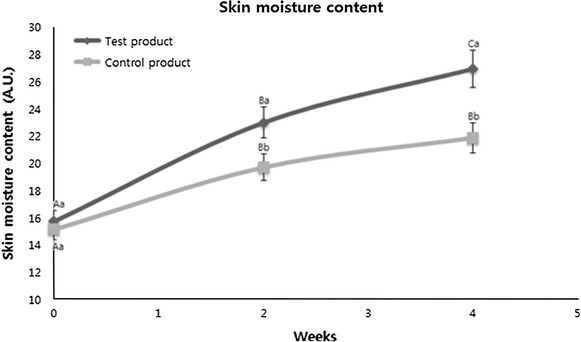
The changes of skin moisture content of the patients. Mean values ±SD from separate triplicate experiments are shown. Means with different letters (*A*–*C*) within same sample are significantly different at *p* < 0.05, and means with different letters (*a*–*b*) within the same period are significantly different at *p* < 0.05.

**Fig. 2:**
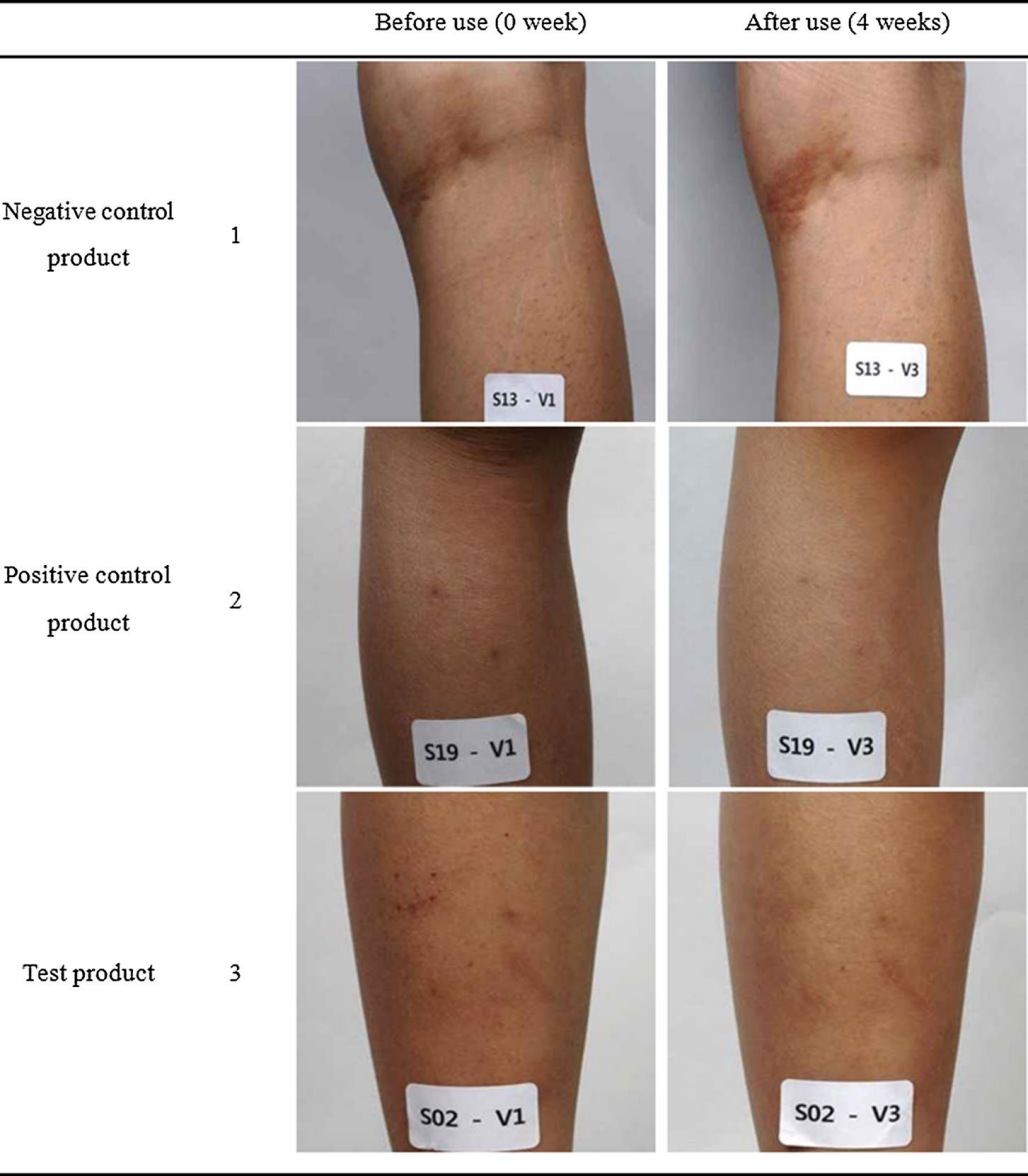
Comparison of the skin of the legs of the patients before and after 4 weeks of treatment with the negative control product (*1*), the positive control product (*2*) and the test product (*3*).

The results of observing the changes in skin water content after using the clinical formulation are shown in Fig. 1 by comparing those from the control product (a positive control using a commercial product). The results from the negative control did not show any improvement even after 4 weeks treatments. (Therefore, any data were not shown in this wok except for the visual comparison in Fig. 2).

Upon reviewing the results, it can be observed that the skin water content increased at 2 and 4 weeks of use of the products compared with before using the products in the group that used the clinical trial formulation and the control group. In the case of the test formulation, the water content increased by 51.85% after 5 weeks of use compared with before using the formulation, and it increased by 79.44% after 4 weeks of use. Given that in a previous study of the effects of oriental medicinal materials on atopic dermatitis the skin water content increased by approximately 21% 6 weeks after use compared with before using the materials, the photosynthetic bacteria clinical formulation used in this study may be judged as being effective for water content (Kang et al. 2009). In the control group, the water content increased 36.62% after 2 weeks of use and 51.18% after 4 weeks of use. Compared with the control group, the group that used the clinical trial formulation had 15.23% higher water content after 2 weeks of use and 28.26% higher water content after 4 weeks of use. Although both the control product and the photosynthetic bacteria clinical trial formulation included materials helpful for skin water content as part of materials needed for their formulation, given that these materials are generally necessary for cosmetic formulation and the differences between the two are remarkable, it can be judged that the clinical trial formulation is much more effective for skin water content than existing products.

### Measurement of the amount of trans-epidermal water loss

To determine the skin water content, the amount of trans-epidermal water loss was measured, and the results are shown in Table 3. In (A) is shown the amount of water loss after 2 and 4 weeks of use compared with before use of the products in the clinical trial formulation and control groups. As with skin water content, the effects were more remarkable in the clinical trial formulation group compared with the control group. In previous atopic dermatitis-related clinical studies in which the effects of glucan and ceramide were examined, the water content decreased by 13–17 after 4 weeks of use, which is similar to the values of 14–15 found in this study; thus, the effects could be confirmed (Yu et al. 2004). After 2 weeks of use, the clinical trial formulation group showed a trans-epidermal water loss rate of change of 15.81%, while the control group had a rate of change of only 5.50%. After 4 weeks of use, the clinical trial formulation group demonstrated a decrease in the amount of trans-epidermal water loss of 27.82% compared with before using the formulation, while the control group demonstrated a lesser decrease in the amount of trans-epidermal water loss of 10.49%. The above results indicate that, compared with existing products, the clinical trial formulation is effective for skin water retention because it prevents trans-epidermal water loss more effectively.

**Table 3:** Measurement of the trans-epidermal moisture loss in the skin

Period (weeks)	Test product (g/h m^2^)	Control product (g/h m^2^)
(a) Trans-epidermal moisture loss		
0	20.31 ± 7.38^Aa^	19.45 ± 6.84^Aa^
2	17.10 ± 6.64^Ba^	18.38 ± 5.52^Bb^
4	14.66 ± 7.31^Ca^	17.41 ± 5.40^Bb^
(b) The percentage of the trans-epidermal moisture loss changes from the zero day of the treatment		
2	15.81%	5.50%
4	27.82%	10.49%

Mean values ±SD from separate triplicate experiments are shown. The means with different letters (A–C) within the same sample are significantly different at *p* < 0.05, and means with different letters (a–b) within the same period are significantly different at *p* < 0.05.

### Changes in the temperature and pH of the skin

Because there was a previous study indicating that if skin surface temperatures increase, the amount of trans-epidermal water loss also increases (Min et al. 1996), the skin temperature was determined and compared with the clinical trial formulation treatment time to further verify the trans-epidermal water loss. The test subjects’ skin temperatures decreased after 4 weeks of treatment with the formulation (Table 4). In the case of the clinical trial formulation group, the relevant change rates were 0.24% after 2 weeks of use and 3.37% after 4 weeks of use, indicating insignificant effects on temperature reduction. In a previous study in which skin temperature was measure to determine the effects of an existing oriental medicinal cosmetic material made from a golden thread detoxifying decoction, the oriental medicinal cosmetics material did not affect skin temperature (Yun et al. 2008). Skin temperature is considered to vary greatly among individuals. Similarly, in the case of the control group, there were almost no temperature reduction effects with a change rate of 0.43% after 2 weeks of use and a change rate of 2.86% after 4 weeks of use. Although the temperature reduction effects of the clinical trial formulation were greater compared with the control group, the differences were small. Therefore, the clinical trial formulation was concluded to have almost no skin temperature reducing effect.

**Table 4:** Measurement of the skin temperature upon treatment with the topical cream

Period (weeks)	Test product (°C)	Control product (°C)
(a) Skin temperature		
0	30.46 ± 1.19^Aa^	30.44 ± 0.84^Aa^
2	30.38 ± 7.14^Aa^	30.31 ± 0.74^Aa^
4	29.41 ± 1.04^Aa^	29.57 ± 0.90^Aa^
(b) The percentage of skin temperature changes from the zero day of the treatment		
2	0.24%	0.43%
4	3.37%	2.86%

Mean values ±SD from separate triplicate experiments are shown. Means with a different letter (A) within the same sample are significantly different at *p* < 0.05, and means with a different letter (a) within same period are significantly different at *p* < 0.05.

**Fig. 3:**
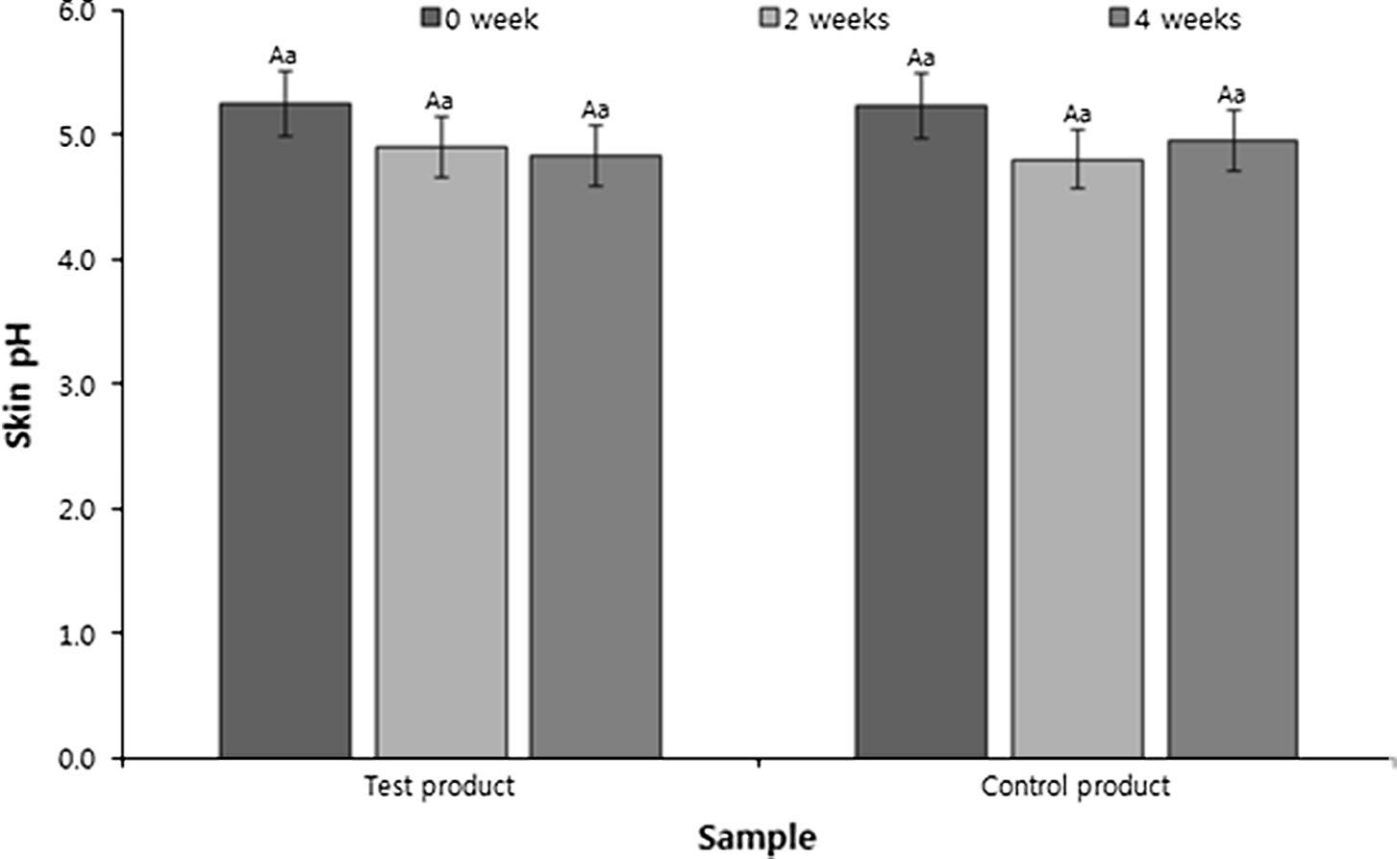
Comparison of skin pH changes for treatment with the cream. Mean values ±SD from separate triplicate experiments are shown. Means with a different letter (*A*) within the same sample are significantly different at *p* < 0.05, and means with a different letter (*a*) within the same period are significantly different at *p* < 0.05.

Based on a previous study (Oh et al. 2010) indicating that younger and healthier skin should have pH levels closer to slight acidity, and skin with atopic dermatitis are alkaline due to damage to skin barriers, skin pH measurement experiments were conducted to further validated the skin water-retaining effects of the clinical trial formulation. Changes in the pH levels appearing in the clinical trial formulation and control groups could be identified until after 4 weeks of use for the formulations (Fig. 3). The pH levels decreased after 2 and 4 weeks of use in the clinical trial formulation group but decreased after 2 weeks of use and increased after 4 weeks of use for the control group. In a previous study in which clinical tests of cosmetics comprising oriental medicinal materials for atopic dermatitis were conducted, the skin pH level increased from 5.4 before treatment to 5.6 after treatment; thus, the skin became slightly acidic (Kang et al. 2009). Similar to that study, although the difference between the clinical trial formulation and control groups was small, the clinical trial product maintained slight acidity of the skin with a pH close to 5 and could have good effects against atopic dermatitis that shows alkalinity.

### Evaluation of the pruritus VAS and SCORAD index

**Fig. 4:**
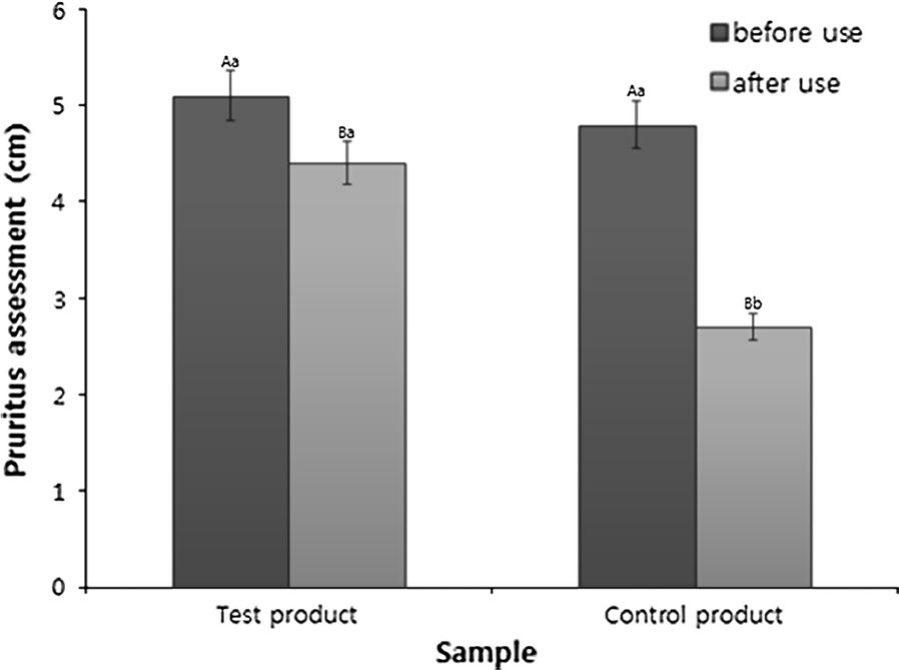
The assessment of the pruritus index of atopic dermatitis patients. Mean values ±SD from separate triplicate experiments are shown. Means with different letters (*A*–*B*) within the same sample are significantly different at *p* < 0.05, and means with different letters (*a*–*b*) within the same period are significantly different at *p* < 0.05.

VAS evaluation was conducted by having the test subjects mark their degree of pruritus using a 10 cm-long line as a pruritus scale. Fig 4 shows that, for of the formulation used in the clinical trial, the pruritus felt by the test subjects that was 5.1 cm on the pruritus scale before using the formulation, and it was reduced to 4.4 cm by 0.7 cm when the formulation was used for 4 weeks. However, the general control product showed a change of 2.1 from 4.8 cm at the beginning to 2.7 cm after 4 week s of use. Therefore, it can be observed that the pruritus reducing effect of the clinical trial formulation was smaller compared with the control product.

The SCORAD index, which enables determining the severity of dermatitis, was evaluated for the test subjects to identify the effects of the clinical trial formulation (Sung et al. 2014). The SCORAD index values after treating the clinical trial formulation and positive control sample were estimated as ca. 20 at 2 weeks of the treatment, and at 4 weeks of the treatment they were decreased further down to 17.05 and 16.95 for the clinical trial formulation and the positive control groups, respectively, but the difference was not significant between two groups. It could tell that about 21% improvement could be observed after 4 weeks treatment of both samples in terms of SCORAD index, compared to the starting day (Table 5b). Another study verifying the effects of cosmetics made of oriental medicinal materials reported that the SCORAD index value after applying cosmetics made of oriental medicinal materials for 6 weeks was 15, which is not much different from the value we found in this study although the application period of the product was 2 weeks longer than the period in this study (Kang et al. 2009). Because the SCORAD index decreased over time after applying the product to the test subjects, the atopic dermatitis-relieving effects of the clinical trial formulation were examined, but we could not conclude that the effects were better compared to existing products.

**Table 5:** Evaluation of the SCORAD index after treatment with the topical cream

Period (weeks)	Test product	Control product
(a) SCORAD index		
0	21.76 ± 6.07^Aa^	21.59 ± 8.24^Aa^
2	20.00 ± 7.14^Ba^	20.00 ± 7.87^Ba^
4	17.05 ± 8.00^Ca^	16.95 ± 7.60^Ca^
(b) The percentage of SCORAD index changes from the zero day of the treatment		
2	8.09%	7.36%
4	21.65%	21.49%

Mean values ±SD from separate triplicate experiments are shown. Means with different letters (A–C) within the same sample are significantly different at *p* < 0.05 and means with a different letter (a) within same period are significantly different at *p* < 0.05.

### Photographic observation of the atopic dermatitis regions before and after the treatments

**Fig. 5:**
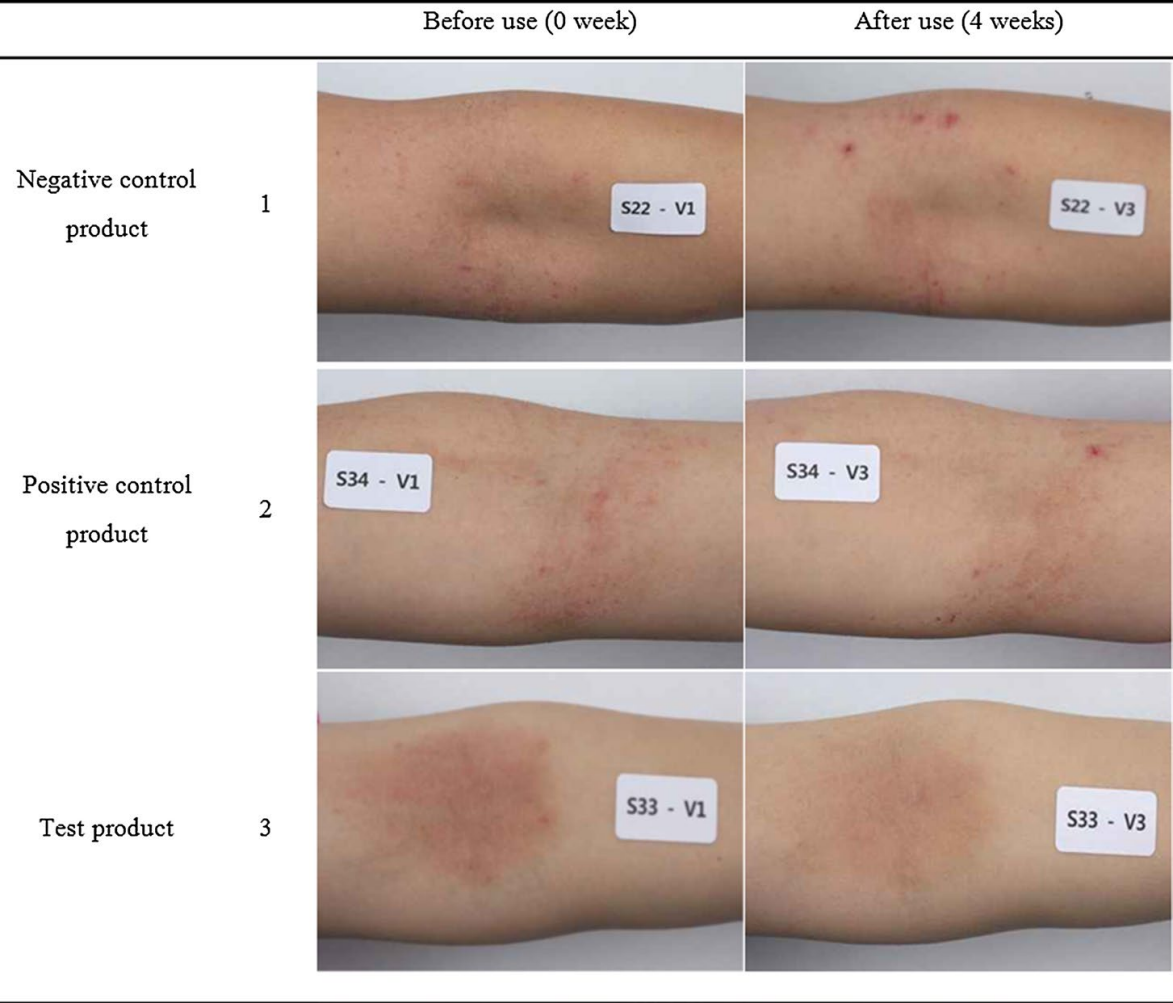
Comparison of the skin of the arms of the patients before and after 4 weeks of treatment with the negative control product (*1*), the positive control product (*2*) and the test product (*3*).

The pictures taken from the same areas of the patients before and after the treatments are shown in Figs. 5 (arms), 2 (legs). In a negative control (1 in Figs. 5, 2), there was no improvement observed even after 4 weeks treatment. However, great improvement was observed for both legs and arms after 4 weeks treatment in using the test products even though there was some enhancement was also observed in treating the control products (commercial products), and there was particularly better efficacy in the legs, which was possibly due to the relatively soft skin of the inner portions of the arms. It can be observed that xeroderma and inflammation were relieved after using the formulation for 4 weeks, and the skin became clear for many patients. The skin of the leg in Fig. 2 also shows that the atopic dermatitis symptoms were relived; thus, the effects of the formulation used in the clinical trial could be identified. Therefore, these results could tell that the effects of the test product on soothing atopic dermatitis were found to be better than those of the commercial product used in this work. This clinical efficacy of the topical cream also seems to be similar to or even better than those shown by other cosmetic containing the extracts from snail mucus (Oh et al. 2010).

## Discussion

In this study, the efficacy of the extracts from photosynthetic bacteria for relieving atopic dermatitis was clinically demonstrated for the first time in the form of a topical cream. The topical cream containing 10% (v/v) extract demonstrated more beneficial effects for skin water content and the amount of trans-epidermal water loss. This result also indicated that this unique formulation could relieve xeroderma, which is a general symptom of atopic dermatitis, and it may also be effective for treating atopic dermatitis. Together with maintaining large skin water content, this cream was capable of maintaining relatively low skin temperature, which could reduce the feeling of itch in the skin. The pH of the skin was also maintained as slightly acidic with the treatment with this cream because healthier skin is closer to being slightly acidic and the potential of the formulation as a countermeasure against atopic dermatitis, which is characterized by alkaline skin, was reported (Yun et al. 2008). From these results, it appears that topical creams containing the extracts from photosynthetic bacteria should be effective for treating or at least soothing atopic dermatitis although some of the formulation chemicals (glycerin, Jojoba oil, SC-glucan) in Table 1 could possibly affect skin water content retention and skin protection. However, the amount of these chemicals is small enough to be negligible for influencing efficacy compared with other commercially available cosmetics. With regards to the SCORAD index of this trial, the values decreased over time after the use of the topical cream, and these results are similar to or even better that that of existing cosmetics.

Based on the above results of pH and water contents changes as well as SCORAD index, it could tell that the effects of the test topical cream for relieving atopic dermatitis may be attributed to the removal of reactive oxygen species (ROS) in the skin, which is a major cause of inflammation by the super oxide dismutase (SOD) and catalase produced by photosynthetic bacteria (Cho and Choi 2013). Therefore, it is very positive that the photosynthetic bacteria formulation in this cream showed remarkable enhancement, compared to those of commercially used topical cream for atopic dermatitis. However, interestingly enough, the VAS scales for pruritus felt by the patients did not improve much in the clinical trial compared with existing cosmetics, which should also be considered when applying this cream to a broad spectrum of patients.
